# Three Surgical Cases

**Published:** 1867-07

**Authors:** Hiram Wanzer

**Affiliations:** Chicago


### THREE SURGICAL CASES.

**Read before the Chicago Medical Society.**

By Hiram Wanzer, M.D., Chicago.

Case I. I was called, the 10th November last, to Mrs. S., German, aged 66. Thirty-six hours previously she had fallen into a cellar, producing an oblique fracture of the humerus, at the surgical neck. The structures of the arm were much swollen and ecchymosed, as also the left mamma and axillary space. The fracture being reduced, a simple form of dressing was used consisting of three well-padded wooden splints for the arm. The fore-arm was supported by a sling, but the elbow was left dependent. The splints were allowed to remain until ossific union occurred, which was between the sixth and eighth weeks, much sooner than we anticipated, on account of her age and a violent cough with which she was afflicted. In order to insure perfect consolidation of the fracture, three pasteboard splints were now applied, and were worn six weeks longer. The callus at the axillary border of the shaft of the humerus was very abundant, but is now nearly absorbed, leaving but a slight protuberance at the seat of fracture. There is an inch shortening of the arm, but there is no obliquity or deviation in the axis of the limb. All the motions of the arm are perfect, except the backward movement, which is limited. We recently had occasion to visit the patient, not having seen her for several weeks, and found, upon inspecting the arm over the brachial, at its lower fourth, a soft, painless, pulsating tumor, the size of a hickory nut. Its entire disappearance upon compressing the artery above the tumor, led me to believe that it was no abnormal deposit in the soft, lax tissues, but that, probably, the coats of the artery were degenerate and involved in traumatic lesion, and that, unmodified by treatment, the case might eventuate in alarming aneurism. I sincerely hope our fears are groundless, for she could hardly survive an accident more grave than the first coming in her winter.

Case II. I was called to Mrs. M., German, aged 30, the 18th January last. She stated that she had been suffering three yeare from leucorrhœa, also from some abnormal growth in the vagina, rendering coition difficult and exceedingly painful. During this time, she had consulted empirics, and also a number of respectable physicians, without relief.

Upon examination, I found the posterior and lateral walls of the vagina studded with flattened condylomata, or warts, which considerably occluded the passage. Anticipating the disease of specific origin, my future treatment was both constitutional and local. The following pills were first given:—℞. Hydrarg. bichlorid. gr. 1½, opii pulvis gr. 6. Div. pill No. 12. S. One pill to be taken in the morning and at bedtime. The pills were continued until slight ptyalism ensued, when a ferruginous tonic was substituted, enjoining rest and nutritious diet. A fruitless attempt was made, to remove the morbid mass by the following local remedies, using each separately in solution, *viz.*:—Argent nitrat., chromic acid, liquor ferri subsulphatis, zinci chloridi, and, finally, the acid nitrate of mercury. The last remedy gave her such increased suffering that she would allow but a single application. Excepting the last two agents used, the warts appeared to prosper and grow by the local stimulation, undergoing but little change under treatment.

Despairing of curing her under any constitutional or local measure adopted, I suggested an operation for their removal, which was assented to. Dr. N. W. Wilber having given chloroform to anæsthesia, I removed the intruding mass with the curved scissors. In consequence of the vascularity of the parts, the hemorrhage was abundant, much confusing the operation. Their entire removal did not completely arrest the leucorrhœa, which consisted of glossy, ropy mucus, not unlike the white of eggs. After the parts were healed by granulation, the speculum revealed the discharges proceeding from the cavity of the uterus; the os uteri was found slightly eroded and inflamed.

A few weekly applications were made of the solid nitrate of silver to the inflamed os and cavity of the uterus, assisted by daily injections of zinci sulphas ʒj, aqua pura ℥xvj., continuing the internal use of tinct. ferri muriatis, 15 drops, three times a day. The local remedies were omitted during the menstrual period. Under the last treatment, her general health and vitality has much improved, the leucorrhœa almost arrested, the inflammatory action of the os and cervix uteri modified, and we anticipate an early cure. She is now attending to her household duties.

I ascribe no honor to myself in the primary treatment of this case, for I believe much valuable time was wasted by not operating in the onset.

Case III. I was called, March 16th, to J. S., a lad 9 years old. He had fallen 10 feet, through a hatchway, alighting upon his head, and was taken to his home in a state of profound insensibility. There was scarcely any pulse at the wrist when I arrived, and the heart's action was nearly suspended; there was general pallor of the countenance, and coldness of the extremities; his every appearance indicated early dissolution. I immediately raised the body and legs to an angle of 45 degrees, and, in less than 15 minutes, quite a full pulse returned at the wrist, the action of the heart was stronger, and the glow returned to the cheeks, showing that the capillary circulation was being established. He was now placed in the recumbent posture, to remove his clothing and examine his body for injuries, and in one moment the pulse forsook the wrist and the heart's action was again more imperceptible. I thought he was gone. I placed him, instantly, in the former position, and, in less than 5 minutes, the pulse returned at the wrist, the heart began to beat again with renewed vigor, and the brain responded to its accustomed stimuli. The *rationale* of the treatment—the blood returned to the capillaries and the machinery of life, temporarily suspended, was again set in motion. He remained in this position 30 minutes longer, when he was placed in the recumbent posture. Cold applications were now applied to the head and hot epithems to the feet, and an enema of whiskey, ℥ss., with gum camphor, gr. v., was given. This measure assisted in completing the reaction. In about 30 minutes from this time, he awoke and laughed. His convalescence was exceedingly rapid, but he was speechless 24 hours, when his voice returned.

I would state that I have no fitting language to express to the Society the gratitude of my heart for the priceless discovery made here, of the therapeutical advantage of *position* in the treatment of shock, and in concussion of the brain. It has amply repaid me for all my attendance here. It has quite recently saved two patients of mine, who, but for it, would have been in the land of spirits.

